# Functionalizing Ferritin Nanoparticles for Vaccine Development

**DOI:** 10.3390/pharmaceutics13101621

**Published:** 2021-10-05

**Authors:** Margarida Q. Rodrigues, Paula M. Alves, António Roldão

**Affiliations:** 1 iBET, Instituto de Biologia Experimental e Tecnológica, Apartado 12, 2781-901 Oeiras, Portugal; margarida.rodrigues@ibet.pt (M.Q.R.); marques@ibet.pt (P.M.A.); 2 ITQB NOVA, Instituto de Tecnologia Química e Biológica António Xavier, Universidade Nova de Lisboa, Av. da República, 2780-157 Oeiras, Portugal

**Keywords:** ferritin nanoparticles, surface decoration, genetic fusion, modular assembly, vaccines, recombinant expression

## Abstract

In the last decade, the interest in ferritin-based vaccines has been increasing due to their safety and immunogenicity. Candidates against a wide range of pathogens are now on Phase I clinical trials namely for influenza, Epstein-Barr, and SARS-CoV-2 viruses. Manufacturing challenges related to particle heterogeneity, improper folding of fused antigens, and antigen interference with intersubunit interactions still need to be overcome. In addition, protocols need to be standardized so that the production bioprocess becomes reproducible, allowing ferritin-based therapeutics to become readily available. In this review, the building blocks that enable the formulation of ferritin-based vaccines at an experimental stage, including design, production, and purification are presented. Novel bioengineering strategies of functionalizing ferritin nanoparticles based on modular assembly, allowing the challenges associated with genetic fusion to be circumvented, are discussed. Distinct up/down-stream approaches to produce ferritin-based vaccines and their impact on production yield and vaccine efficacy are compared. Finally, ferritin nanoparticles currently used in vaccine development and clinical trials are summarized.

## 1. Introduction

Vaccination is one of the most effective tools to prevent infectious diseases. Traditional vaccines are based on attenuated or inactivated organisms/viruses; while the former presents safety risks due to a possible spontaneous reversion, the production process of the latter may denature antigenic peptides and lessen the immunogenicity of the inactivated vaccine [1]. In order to reduce safety concerns or loss of efficacy related to traditional vaccines, research has focused on the development of subunit vaccines, i.e., pathogen-derived antigenic components [2]. Although safer, these are generally less immunogenic than traditional vaccines [1,2]; thus, several strategies have been exploited to improve the immune response of subunit vaccines. Examples include the addition of adjuvants, optimization of delivery systems, or the use of particulate antigen assemblies, in which antigens are attached to particles [2]. In addition, the applicability of subunit vaccines is not restricted to infectious diseases, but may also be used in cancer therapy [3] and treatment of autoimmune diseases [4,5].

When considering subunit vaccination, there is ample evidence that nanoparticle-anchored antigens are more efficacious than monomeric, soluble ones [6]. The affinity between the nanoparticle surface and the cell membrane contributes to a more efficient cellular uptake of the anchored antigens when compared to antigens alone [7]. Nanoparticles of ~100 nm are considered the most favorable for cellular uptake by dendritic cells (DCs) since this size matches those of viruses and bacteria, the natural targets of DCs [8]. These cells are the starting point of the adaptive immune response as they detect pathogens, sample them, and migrate to lymph nodes (LNs) where they present antigens to T- and B-cells thus triggering an immune response [7]. In addition, ordered display/distribution of antigens on the surface of nanoparticles allows multivalent binding of B-cell receptors thus enhancing the interaction with cognate B-cells [9]. The pharmacokinetics of nanoparticle-carried antigens is distinct from that of monomeric, soluble antigens. While the latter are diffused into body fluids and diluted upon administration, nanoparticles allow the maintenance of antigen clusters, and those with size ranges of 20–200 nm are found to have long circulation times [10]. Another benefit of nanoparticles is the likelihood of enhanced transport to LNs, which augments antigen uptake by LN-resident DCs and subsequent activation of immunological responses by T- and B- cells [7]. A further advantage of nanoparticle-based vaccines is the possibility of retention at the injection site, which enhances antigen uptake and presentation by DCs [6].

Natural-derived nanoparticles are biocompatible and hydrophilic, rendering them strong contenders for biomanufacturing purposes. Among these, protein-based nanocages with self-assembling properties such as ferritin and virus-like particles [11] are especially interesting for vaccine development as they mimic both the size and structure of pathogens and are amenable to surface conjugation of antigens to promote the interaction with immune cells [12].

Ferritin can be found in almost all living organisms, including bacteria, fungi, plants, and animals [13]. Its major physiological function is to store iron in an insoluble non-toxic form while keeping it bioavailable intracellularly by converting it to its soluble form [14], having an important role in iron homeostasis. It also provides a protective effect against toxicities involving free iron, such as the generation of reactive oxygen species which can damage cellular machinery and lead to cell death [15].

Ferritin has recently emerged as a promising platform for antigen display [16]. In addition to its self-assembly capacity, the ferritin protein complex presents remarkable thermal and pH stability (withstanding temperatures up to 80–100 °C and pH ranges of 3–10), monodispersity, small uniform size, biocompatibility, biodegradability, low-cost large-scale production, hollow cavity with reversible assembly/disassembly, and amenability to surface conjugation by chemical or genetic approaches [17]. Besides vaccine development, ferritin has been used in nanobiotechnology for drug delivery, biomimetic synthesis, bioimaging, and cell targeting [17,18,19,20,21].

**Figure 1:**
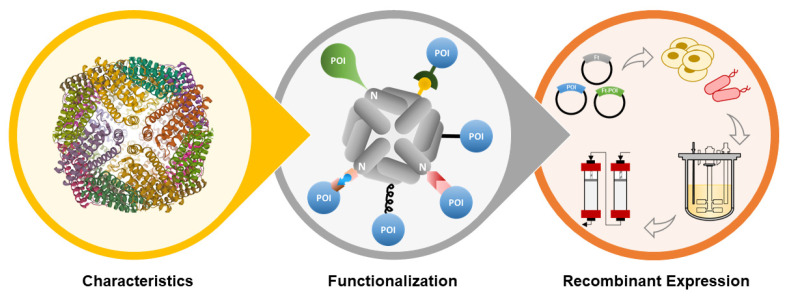
Schematic overview of the topics discussed in this review: ferritin characteristics, functionalization, and recombinant expression of ferritin nanoparticles for antigen-display applications.

In this review, the properties of ferritin nanoparticles (structure, assembly mechanism, and glycosylation profiling) will be briefly described, followed by a thorough review of (i) bioengineering strategies for ferritin nanoparticles functionalization, and (ii) up/down-stream processes and analytics used for ferritin nanoparticles production and characterization, all in the context of vaccine development (Figure 1).

## 2. Ferritin Properties

### 2.1. Structure

Ferritin nanoparticles ubiquitously found in nature contain a hollow core of inner and outer diameters of 8 and 12 nm, respectively, that can internalize up to 4500 iron atoms in the form of ferric oxyhydroxide [22], with variable amounts of phosphate [23]. The ferritin structure of mammalians, amphibians, plants, and bacteria has been reviewed elsewhere [24]. Although there are considerable variations in the amino acid sequence (up to 80%) [23] and the presence of haem moieties in some bacterial ferritins [24], their tertiary structure is quite similar and some subdomains such as the iron entry/exit channels contain highly conserved sequences across different species [23]. Ferritin particles isolated from vertebrates are composed of two types of subunits, H-chain (heavy, 21 kDa) and L-chain (light, 19 kDa), whereas those found in plants and bacteria contain only one type resembling the H-chain of vertebrates [24].

**Figure 2:**
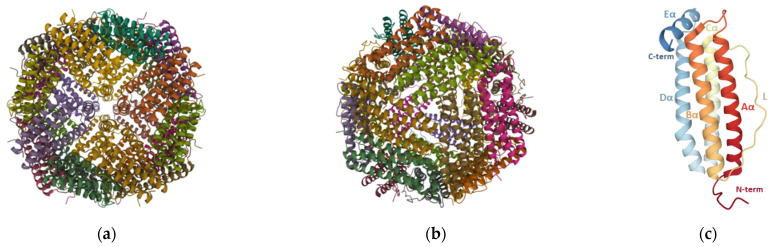
Native ferritin structure. Quaternary structure of human L-chain ferritin (PDB ID: 2FFX [26]), consisting of 24 subunits, assembled in octahedral (432) symmetry around the (**a**) 4-fold and (**b**) 3-fold axes. (**c**) Tertiary structure of human L-chain ferritin with labeled motifs: A-to-E-α helices, long (L) loop, and C- and N-terminals (C-term and N-term, respectively). Images adapted from the RCSB PDB (rcsb.org) [27] of PDB ID: 2FFX [26] created with Mol * viewer [28].

Each ferritin particle is made up of 24 identical or homologous subunits that self-assemble in octahedral (432) symmetry such that small channels are formed at the 4-fold and 3-fold symmetry axes (Figure 2a,b). These channels allow the passage of iron and other ions or small molecules [22], with iron being guided via the 3-fold channels [25]. Each monomer is composed of a four-α-helix bundle (A, B, C, and D helices) with a short fifth E-α-helix at the C-terminus (Figure 2c) that runs from the outside to the inside, making the C-terminal end of the ferritin chain to be inside the assembled nanoparticle. Additionally, a long loop (L) of about nineteen residues connects the C-terminal of B-helix to the N-terminal of C-helix. The N-terminal, L loop, and A- and C-helices are solvent-accessible, while the C-terminal, and B- and D-helices face the inner side of the ferritin nanocage [24].

### 2.2. Assembly Mechanism

The unraveling of supramolecular structures’ self-assembly mechanism is challenging, and the symmetrical and nearly homo-oligomeric characteristics of ferritin nanoparticles contribute to making it even more arduous to decipher.

X-ray diffraction experiments conducted on horse spleen apoferritin suggested that stable dimers are the first intermediates in self-assembly, as dimers interact along most of their length and have a larger mutual area of contact than those around the 3- or 4-fold axes [29]. The second step of the assembly was hypothesized to be the formation of hexamers by aggregation of dimers around the triagonal axis [29] because, compared to the 4-fold axis, the contact region around the 3-fold is greater. In addition, the contact between subunits around the 4-fold pore is tenuous (lined by the short E-helices) thus making it unlikely for stable symmetrical tetramers to occur. 

Gerl and Jaenicke [30] were the first to propose the self-assembly mechanism of ferritin when the reconstitution of ferritin monomers of horse spleen apoferritin was monitored through chemical cross-linking and subsequent spectroscopic analysis. The overall proposed mechanism is described by Equation (1):

(1) $$24\;m_{1}\to 24M_{1}⇌8\;M_{1}+8M_{2}⇌8M_{3}⇌4M_{6}⇌2M_{12}\to M_{24}$$

More recently, Sato et al. [31] studied the assembly of the homopolymeric *Escherichia coli* ferritin A using time-resolved small-angle X-ray scattering (TR-SAXS). Differently from the hetereopolymeric horse spleen ferritin, monomers and trimers were reported to be unlikely intermediates during the assembly of homopolymeric ferritin. Sato et al. [31] explained the assembly of ferritin by a simple model in which only tetramers, hexamers, and dodecamers were considered as intermediates:

(2) $$12M_{2}⇌6M_{4}⇌4M_{6}⇌2M_{12}⇌M_{24}$$

Although the SAXS profile is consistent with the work developed by Gerl and Jaenicke [30] (ferritin assembly follows a second-order reaction and is based on the association of oligomers), the simplified model presented in Equation (2) had some limitations such as being concentration-dependent and unable to identify all possible intermediates according to SAXS data.

### 2.3. Glycosylation Profiling

There is evidence that native ferritin can undergo post-translational modifications, including change in isoelectric point [32], phosphorylation [33], and glycosylation [34]. Physiologically, most of the synthesized ferritin remains within the cell where it is responsible for maintaining the intracellular iron homeostasis. There, both H- and L-chains of human ferritin are unglycosylated, and their ratio varies between different cell types and maturation stages. Ferritin can also be found in the plasma consisting mainly of *N*-glycosylated L-chain [34] that may be sialylated [35]. Glycosylation may influence the removal rate of ferritin from the plasma by hepatocytes, the major cell type responsible for clearing plasma ferritins. These cells enclose in their membrane a specific receptor for both glycosylated and non-glycosylated ferritin [36], and there is evidence that glycosylated ferritin is cleared at a slower rate than non-glycosylated ferritin, determining a significantly longer half-life for the glycosylated ferritins [34]. 

Microbes (e.g., *E. coli*) and animal cells (e.g., human embryonic kidney, HEK293; Chinese hamster ovary, CHO; and *Sf*-9, a clone isolate of *Spodoptera frugiperda Sf*-21) have been used as expression hosts, and thus the resultant recombinant ferritin presents distinct glycosylation profiles (Table 1). Most laboratory-produced ferritin is unglycosylated. This results from either (i) using expression systems that are unable to perform glycosylation (*E. coli*), (ii) production of ferritin subunits that miss a putative *N*-glycosylation consensus sequon (e.g., some bacterial ferritins, insect H-chain ferritin), (iii) production of ferritin chains containing a putative *N*-glycosylation consensus sequon but that is sterically hindered from being glycosylated (e.g., H-chain ferritin of mammalians), or (iv) introduction of point mutations to avoid potential *N*-glycosylation sites (e.g., *Helicobacter pylori* (N19Q) and *Rana catesbeiana* (N8Q)). The latter is distinct from the others since ferritin is produced in its unglycosylated form through genetic engineering. In this case, suppressing glycosylation sites on ferritin avoids glycan-induced steric hindrance or undesired interactions between the glycan and chemical groups on antigens of interest (AOI) that may be fused to the ferritin; that else could prevent the antigen from acquiring its natural tertiary structure, and thus its immunogenic potential is at an impasse.

Other mutations have also been performed to improve the use of ferritin nanoparticles in nanobiotechnology: (i) the point mutation R64K in *Pyrococcus furiosus* ferritin eliminated a potential cleavage site [9], (ii) ligands with affinity for thiol groups were conjugated to engineered ferritin mutants from *Archaeoglobus fulgidus* and *P. furiosus* bearing cysteine point mutations in selected topological positions [54], and (iii) the *H. pylori*-bullfrog hybrid carrying the N-terminal 2–9 residues from the bullfrog *R. catesbeiana* L-chain ferritin (N8Q) allowed the formation of radially projecting tails, improving antigen presentation in many ferritin-based vaccine candidates [48,49,50,51,52,53].

Conversely, the conjugation of a trimannose moiety to ferritin nanoparticles led to a pronounced accumulation of these particles on follicular DCs (i.e., LN-resident DCs) within 3 days of immunization in mice, whereas bare ferritin nanoparticles lacking glycosylation showed low overall accumulation in LNs and no colocalization in follicular LNs [9]. Hence, the presence of even simple glycans on ferritin could be beneficial, potentially directing vaccine nanoparticles to the follicular LNs network and prolonging the half-life of ferritin-based vaccines upon administration.

## 3. Functionalization of Ferritin Nanoparticles

Ferritin nanoparticles contain three distinct exploitable interfaces: the interior and external surfaces, and the intersubunit regions. All are amenable to manipulation through chemical and genetic engineering to render them useful nanocages in biotechnology. 

Intersubunit modifications have been performed in ferritin, especially to modify the nanoparticle assembly/disassembly mechanism. Usually, ferritin subunits assemble spontaneously upon expression. The disassembly process can be induced under extremely acidic (pH < 2) or basic conditions (pH > 10), or in the presence of reducing agents [19]; the reassembly occurs when the subunits are back to neutral conditions. Huard et al. [76] redesigned the interface of ferritin subunits so that their interaction could be chemically induced by metal-binding; the authors were able to coordinate ferritin self-assembly via selective response to Cu(II) at neutral pH. In a different study [77], the interface of ferritin subunits was genetically modified to yield nanocages capable of disassembly at pH 4.0 and reassembly at pH 7.5. The engineering of ferritin to yield an assembly/reassembly mechanism that occurs at mild conditions is relevant when the nanoparticle is exploited to encapsulate pH-sensitive molecules, such as some peptides and drugs. 

**Figure 3:**
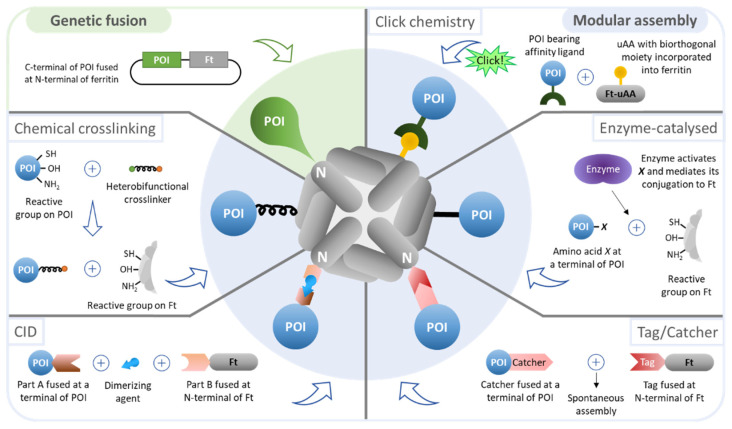
Technologies used for the design of functionalized ferritin nanoparticles. The outer surface of ferritin (Ft) nanoparticles can be functionalized with a protein of interest (POI) by genetic fusion (green-shaded) and modular assembly (blue-shaded). CID—chemically inducible dimerization.

The hollow cavity of ferritin can be engineered to encapsulate and transport several molecules with distinct purposes, including peptides, drugs, imaging agents, and polymers [14]. In addition, it can also act as a size-constrained reaction vessel for nanomaterial synthesis [18]. By adding targeting moieties to the external surface of ferritin, the encapsulated molecules can be delivered in a site-specific manner. Additionally, the outer surface of ferritin can be engineered to impart increased circulatory half-life (e.g., PEG [69], PAS polypeptides [70]) and, most importantly, to trigger specific cellular responses (e.g., antigens, antibodies, nucleotides) [14]. In the following sections, functionalization of the outer surface of ferritin will be thoroughly described since this interface enables antigen presentation for the development of ferritin-based vaccines (Figure 3).

### 3.1. Bioengineering Strategies

#### 3.1.1. Genetic Fusion

Delivering antigens anchored to a scaffold can be achieved by genetic fusion of the gene encoding the AOI to the gene encoding the protein scaffold. After expression, self-assembly of the protein scaffold subunits results in multimerization and ordered display of antigens. 

For efficient presentation and interaction with cellular receptors, it is crucial that antigens retain their structural stability and conformation. However, it is challenging to recombinantly express antigens fused to a scaffold without impairing their stability or conformation, and consequently their activity. For instance, the antigen may interfere with intersubunit interactions of the protein scaffold, hindering its assembly into nanoparticles. Since the chimera does not occur in nature, and often its components derive from distinct cell types and/or cellular locations (with different and sometimes incompatible properties such as pI, charge, and hydrophobicity), genetic fusion may cause improper folding of protein scaffold subunits and/or antigens resulting in either inhibition of scaffold assembly or loss of the antigen activity. In addition, the optimal host for protein scaffold expression may not be optimal for AOI expression, or vice-versa.

Since Li et al. [16] functionalized the outer surface of ferritin with a Tat peptide from HIV-1, many other studies have used ferritin as an antigen delivery system. From single peptides with a dozen residues in length to complex trimers, several antigens have been successfully expressed in combination with ferritin to generate valuable nanoparticles for vaccine development and/or cell targeting applications (summarized in Table 2).

One example is the work of Kanekiyo et al. [45], in which trimeric haemagglutinin (HA) was introduced into the 3-fold axis of the ferritin nanoparticle resulting in the generation of particles with eight trimeric viral spikes on their surface. By fusing the HA gene to the N-terminal of ferritin, which is opportunely located around the 3-fold axis, HA monomers were in proximity to interact with each other, resulting in reciprocal stabilization and allowing for the oligomerization of conformation-dependent trimeric antigens. The ferritin-HA nanoparticles not only acquired the desired physical properties (final product homogeneity, native conformation, and symmetric display) but also enhanced the immunological potency, compared to the licensed inactivated vaccine—the nanoparticle vaccine-elicited HA antibody titters over 10-fold higher in immunized mice. The promising results obtained with this design have motivated the development of three vaccines against influenza, to be tested in Phase 1 clinical trials (ClinicalTrials.gov ID NCT03186781, NCT03814720, and NCT04579250) [45,46]. Results are only available for trial NCT03186781, in which a 60-µg dose in a double vaccine administration regimen, alone or prime-boosted with an influenza DNA vaccine (4 mg), resulted in seroconversion rates of 40% or 90%, and 50% inhibitory concentration (IC_50_) titers of 1 × 10^3^ or 3 × 10^3^, respectively. No significant adverse effects were detected.

The approach by Kanekiyo et al. [45] has also been used to functionalize ferritin with HA derived from different influenza strains in search for a “universal” influenza vaccine [46,51,52], the trimeric envelope glycoprotein from HIV-1 (gp140) [9,63,78,79,80], and more recently, the spike (S) protein of the severe acute respiratory syndrome coronavirus 2 (SARS-CoV-2) [81,82,83]. One of these vaccine candidates was reported to elicit rapid immune responses after a single immunization and being highly protective in a mouse challenge model and is now currently been assessed in a Phase 1 clinical trial (ClinicalTrials.gov ID NCT04784767) [83].

Besides the influenza and SARS-CoV-2 vaccines mentioned above, only one other ferritin-based vaccine candidate is in a Phase 1 clinical trial (ClinicalTrials.gov ID NCT04645147) against the Epstein-Barr virus (EBV), causative of infectious mononucleosis and associated with various cancers and autoimmune diseases [50]. As with most ferritin-based vaccines reported in the literature, each ferritin subunit is fused to the AOI, in this case, a subdomain of the envelope glycoprotein 350/220 (gp350) of EBV, so that each ferritin nanoparticle displays twenty-four gp350 epitopes in an orderly and symmetrical fashion [50]. Compared to soluble gp350, the neutralization capacity of the gp350-ferritin nanoparticle increased 10- to 100-fold in immunized mice.

Kim et al. [37] have applied a methodology to increase the solubility of recombinant ferritin nanoparticles in bacterial hosts worth mentioning. Antigens derived from human viruses are prone to the formation of inclusion bodies when expressed in bacterial hosts, and their display in multimeric nanoparticles remains a challenge. In order to improve the production of nanoparticle vaccines in bacterial hosts, RNA was implemented as a molecular chaperone to improve protein folding and enable soluble expression in bacteria. The human RNA-interaction domain (hRID) was fused to the N-terminal of the receptor-binding domain (RBD) of the Middle East respiratory syndrome-coronavirus (MERS-CoV), separated by a TEV cleavage site, while the C-terminal of RBD was fused to the ferritin. Remarkably, the RNA binding capacity of hRID enabled the soluble expression of hRID-RBD-ferritin monomers in *E. coli*, and the following proteolytic removal of the hRID prompted the assembly of monomers into nanoparticles of regular sizes and immunologically relevant conformations.

**Table 2:** Surface functionalization of ferritin nanoparticles by genetic fusion for antigen-display applications.

Expression System	Ferritin Source	Epitope/Antigen	No. Residues	Target	Architectonic Tags	Attachment Region on Ferritin	Yield (mg/L)	Application	Ref
*E. coli*	Human L-chain	Tat peptide	9	HIV-1	Tat peptide and ferritin spaced by a GGG linker	N-terminal	5–10	Viral vaccination	[16]
*E. coli* BL21(DE3)	Human H-chain	RGD4C peptide	9	α_v_β_3_ integrins upregulated on tumor vasculature	RGD4C and ferritin spaced by a GGGT linker	N-terminal	10	Tumor targeting	[68]
*E. coli* BL21(DE3)	*P. furiosus*	FcBP	13	Fc region of antibodies	FcBP and ferritin spaced by G-rich linkers	Loop connecting D and E helices	10	Antibody immobilization	[56]
*E. coli* BL21(DE3)	Rat H-chain	EV71 VP1 peptides	4–15	EV71	-	Loop (res 146) orC- or N- terminal	-	Viral vaccination	[20]
*E. coli* BL21(DE3)	*P. furiosus*	OT-1 OT-2	8 17	T-cell receptors of OT-1 and OT-2	-	Loop (res 146) orC-terminal	-	DC-targeting and vaccination	[57]
*E. coli* BL21(DE3)	*H. pylori*	MtrE loop 1 MtrE loop 2	16 22	*N. gonorrhoeae*	-	Loop connecting A and B helices (res 34) or N-terminal	-	Bacterial vaccination	[38]
*E. coli* BL21(DE3)	Human H-chain	RBM of S protein	69	SARS-CoV-2	RBM and ferritin spaced by (GGGGS)_3_ linker	N-terminal	-	Viral vaccination	[66]
*E. coli* BL21(DE3)	Human H-chain	3x M2e peptide	72	Influenza virus	M2e and ferritin spaced by a GGGGS linker	N-terminal	0.5	Viral vaccination	[72]
*E. coli* BL21(DE3)	Human H-chain	RFP	225	RFP-expressing melanoma tumor cells	RFP and ferritin spaced by a G_3_SG_3_TG_3_SG_3_ linker	C-terminal	-	Tumor targeting and vaccination	[71]
*E. coli* BL21(DE3)	*H. pylori*-bullfrog hybrid	OspA	247	*B. burgdorferi*	OspA and ferritin spaced by GS linker	N-terminal	-	Bacterial vaccination	[49]
*E. coli* Shuffle^®^T7	*E. coli* K12	hRID-RBD of S protein	343	MERS-CoV	TEV cleavage site between hRID and RBD, hRID-RBD, and ferritin spaced by an SSG linker	N-terminal	1.6	Viral vaccination	[37]
*E. coli* BL21(DE3)	*H. pylori*	VP6	410	Rotavirus A	VP6 and ferritin spaced by an SGG linker	N-terminal	-	Viral vaccination	[39]
*Sf*-9	*H. pylori*	GP5	~200	PRRSV	GP5 and ferritin spaced by a GGGS linker	N-terminal	-	Viral vaccination	[41]
*Sf*-9	*H. pylori* (N19Q)	FMDV VP1 G-H loop	211 20	FMDV	VP1 or G-H loop and ferritin spaced by a linker	N-terminal	-	Viral vaccination	[43]
*Sf*-9	*H. pylori* (N19Q)	E2 from CSFV	320	CSFV	E2 and ferritin spaced by a GSG linker	N-terminal	-	Viral vaccination	[44]
HEK293F	*H. pylori*-bullfrog hybrid	RBD of HA	208	Several influenza virus strains	HA and ferritin spaced by an SG linker	N-terminal	-	Viral vaccination	[52]
HEK293T	*H. pylori*-bullfrog hybrid	RBD of S protein	223	SARS-CoV-2	RBD and ferritin spaced by a SSGGASVLA linker	N-terminal	-	Viral vaccination	[48]
HEK293F	*H. pylori*-bullfrog hybrid	gp350 (D_123_)	413	EBV	gp350 and ferritin spaced by an (SG_3_)_2_ linker	N-terminal	-	Viral vaccination	[50]
HEK293F	*H. pylori*-bullfrog hybrid	HA	~500	Influenza virus	-	N-terminal	-	Viral vaccination	[51]
HEK293F	*H. pylori* (N19Q)	HA	550	Influenza virus	HA and ferritin spaced by an SGG linker	N-terminal	2–10	Viral vaccination	[45]
HEK293	*H. pylori* (N19Q)	HA	550	Influenza virus	HA and ferritin spaced by an SGG linker	N-terminal	-	Viral vaccination	[47]
HEK293F	*P. furiosus* (R64K)	MD39	634	HIV-1	MD39 and ferritin spaced by a GSG linker	N-terminal	-	Viral vaccination	[9]
HEK293F	*H. pylori*	gp140	638	HIV-1	gp140 and ferritin spaced by a GSG linker	N-terminal	-	Viral vaccination	[78]
HEK293F	*T. ni* L- and H-chains	gp140	~638	HIV-1	gp140 and ferritin spaced by a (GS)_5_ linker	N-terminal	1	Viral vaccination	[63]
HEK293F	*H. pylori*	S protein	~1148	SARS-CoV-2	S protein and ferritin spaced by a GSGGSG linker	N-terminal	5	Viral vaccination	[83]
HEK293F	*H. pylori*	S protein	1213	SARS-CoV-2	S protein and ferritin spaced by an SGG linker	N-terminal	-	Viral vaccination	[81]
HEK293F CHO	*H. pylori*-bullfrog hybrid	F protein	449	RSV	F protein and ferritin spaced by an S(GS)_2_ES linker	N-terminal	4.7 (HEK) 89 (CHO)	Viral vaccination	[53]
HEK293F CHO	-	E2 from HCV	~500	HCV	E2 and ferritin spaced by a (G_4_S)_2_ linker	N-terminal	0.5 (HEK) 20 (CHO)	Viral vaccination	[84]
HEK293F CHO	-	gp120 gp140	~450 664	HIV-1	gp120 and ferritin spaced by an ASG linker; gp140 and ferritin spaced by a (GS)_5_ASG linker	N-terminal	-	Viral vaccination	[79]
CHO	-	gp140	664	HIV-1	-	N-terminal	-	Viral vaccination	[80]
CHO	-	S protein	1149	SARS-CoV-2	S protein and ferritin spaced by a G_4_S linker	N-terminal	0.8–1	Viral vaccination	[82]

E2 from CSFV: E2 glycoprotein of classical swine fever virus (CSFV); E2 from HCV: E2 glycoprotein of hepatitis C virus (HCV); EV71 VP1 peptides: res 208–222 of capsid protein VP1 from human enterovirus 71 (EV71) identified as important neutralizing epitope of EV71; FcBP: fragment crystallizable (Fc) region of antibodies binding peptide (BP); FMDV VP1: capsid protein VP1 from foot-and-mouth disease virus (FMDV); F protein: fusion protein from respiratory syncytial virus (RSV); G-H loop: main neutralizing antigen site of FMDV VP1; gp120: human immunodeficiency virus 1 (HIV-1) envelope glycoprotein; gp140: HIV-1 envelope glycoprotein (BG505 SOSIP.664); gp350 (D_123_): res 2–415 of the ectodomain of glycoprotein 350/220 from Epstein-Barr virus (EBV); GP5: envelope glycoprotein 5 from porcine reproductive and respiratory syndrome virus (PRRSV); HA: haemagglutinin from influenza virus; hRID: RNA interaction domain of human Lysyl-tRNA synthetase; M2e peptide: M2e amino sequence of HA; MD39: improved version of BG505 SOSIP gp140 from HIV-1; MERS-CoV: Middle East respiratory syndrome coronavirus; MtrE: outer membrane channel of the highly conserved gonococcal MtrCDE active efflux pump from *Neisseria gonorrhoeae*; OspA: lipoprotein on *Borrelia burgdorferi* outer membrane surface when the bacteria reside in the tick gut; OT-1/-2: CD8^+^ and CD4^+^ T cell epitopes corresponding to res 257–264 and 323–339 of ovalbumin, respectively; RBM: receptor binding motif; RGD4C: active peptide targeting the α_v_β_3_ integrins; RBD: receptor binding domain; RFP: red fluorescent protein; S protein: spike protein from severe acute respiratory syndrome coronavirus 2 (SARS-CoV-2); VP6: intermediate capsid protein of human rotavirus A.

#### 3.1.2. Modular Assembly

Nanoparticles generated in more than a single stage (i.e., the nanoparticle and the AOI are produced separately and then conjugated) are referred to as modular assemblies. Compared to genetic fusion, modular assembly adds complexity to the process as the number of production and purification steps increases, yet it bypasses the challenges associated with genetic fusion. Distinct approaches of modular assembly to functionalize the outer surface of ferritin nanoparticles are depicted in Figure 3 and summarized in Table 3.

##### Chemical Crosslinking

The classic approach of the modular assembly comprises chemical conjugation between the nanoparticle and a protein of interest (POI) using crosslinkers that interact with reactive species on the side chains of the proteins. The sulfhydryl group (–SH) of cysteine residues (Cys) is traditionally exploited since this amino acid rarely exists on the surface of proteins and allows site-specificity when a single Cys is available on the protein. The other end of the crosslinker may react with primary amines (–NH_2_), found in lysine residues (Lys) and at the N-terminal of a protein, or, less frequently, with hydroxyl groups (–OH), found in serine (Ser) and threonine (Thr) residues and at the C-terminal of a protein.

Chemical crosslinking to decorate the outer surface of ferritin has been reported in the literature. Falvo et al. [85] conjugated an average of three molecules of IgG per ferritin nanoparticle using a NHS-PEG-Mal crosslinker. Primary amines available on the antibodies were acetylated at the expense of *N*-hydroxy succinimide (NHS), yielding IgG-PEG-Mal, followed by alkylation of Cys on the surface of ferritin by the maleimide arm of the crosslinker. Using a different strategy, Luo et al. [86] functionalized ferritin with KGDS peptide to target human activated platelets. The authors first activated the hydroxyl group at the C-terminal of the peptide with an EDC crosslinker and further functionalized it with sulfo-NHS, yielding KGDS-NHS. Finally, this molecule was conjugated to available Lys on the surface of ferritin at the expense of NHS.

Generally, a protein contains several available primary amines and hydroxyl groups on its surface, thus the final product of chemical crosslinking between proteins is heterogenous and difficult to predict or analyze. Another drawback associated with this method is that Cys residues, added to a genetically engineered protein to offer site-specificity, may interfere with the formation of pre-existing disulfide bonds. To circumvent the challenges associated with chemical crosslinkers, novel approaches of bioconjugation have been explored, which are addressed next.

##### Chemically Inducible Dimerization (CID)

CID uses a small molecule as an intermediate to induce the binding of two different proteins. Compared to a bifunctional crosslinker used in chemical conjugation, it offers higher affinity and specificity, as well as faster kinetics [87]. The FKBP/FRB/rapamycin system has been employed to functionalize the outer surface of ferritin. Ducasse et al. [58] fused FRB to eGFP, and FKBP to a ferritin subunit, and the irreversible attachment of eGFP to ferritin was achieved by the heterodimerization of FKBP and FRB upon addition of the dimerizing agent—rapamycin. Similar to genetic fusion, CID allows for homogeneity of the final product but restricts conjugation to either the N- or C-terminal of the conjugated proteins since the peptides binding to the dimerizer (e.g., FKBP and FRB) are required to be fused at those positions.

##### Click Chemistry

Click chemistry is an alternative method in which unnatural amino acids (uAAs), bearing biorthogonal reactive groups, are incorporated in the polypeptide chain of a protein-based nanoparticle. This type of reaction occurs between functional groups and is characterized by being fast, selective, and having high yields. While this approach offers the advantage of enabling the conjugation of proteins at nearly any site in the uAA-containing protein, it requires artificial amino acid incorporation, metabolic engineering, and additional synthetic steps thus considerably increasing process complexity and cost and hampering scale-up. In addition, the conjugate protein must be functionalized with a ligand with affinity to the uAA. uAAs can be site-specifically incorporated when an amber stop codon in the mRNA is recognized by a tRNA incorporating the uAA. This strategy was used by Khoshnejad et al. [65] to insert the 4-azidophenylalanine (4-AzF) uAA at residue 5 of ferritin. Dibenzylcyclooctyne (DBCO)-functionalized IgG were site-specific conjugated to ferritin nanoparticles by click chemistry between the azide-bearing 4-AzF and the alkyne group in DBCO.

##### Enzyme-Catalyzed Conjugation

Enzyme-mediated conjugation is similar to CID, but instead of using a dimerizer as an intermediate to bind two peptides, it uses a catalyzer (i.e., enzyme) to activate an amino acid residue in a given protein so that it may interact and bind to a second protein. The anti-EGFR nanobody 7D12 was engineered to present a glutamine (Q) residue at the C-terminal, which becomes activated upon addition of transglutaminase and conjugates to a genetically added Lys-Lys at the N-terminal of ferritin [73].

Enzyme-mediated conjugation does not restrict the attachment of a protein to either the C- or N-terminal of ferritin as in genetic fusion and CID approaches. For instance, tyrosinase enables direct conjugation of solvent-exposed tyrosine residues (Tyr) to Cys sulfhydryl groups. Genetically modified endorphin to present Tyr at its terminal was successfully activated and irreversibly conjugated to MS2 viral capsids, each subunit of the 180-mer nanoparticle containing a single native Cys residue at position 47 [88]. However, as aforementioned, the mutation of a protein to add a Cys residue amenable of conjugation may interfere with the formation of native disulfide bonds.

##### Tag/Catcher Technology

Many Gram-positive bacteria contain extracellular proteins with spontaneous intramolecular peptide bonds that confer their high stability in extreme conditions, such as pH 2 and temperatures up to 100 °C. Some of these proteins were engineered to create the Tag/Catcher technology, including the second immunoglobulin-like collagen adhesin domain (CnaB2) from the fibronectin-binding protein FbaB found in invasive strains of *Streptococcus pyogenes*. CnaB2 contains a single isopeptide bond, and by splitting it into peptide and protein fragments followed by rational modification of the parts, a peptide tag of 13 amino acids (SpyTag) can spontaneously form a stable covalent bond with its protein partner (SpyCatcher, 138 amino acids, 15 kDa) [89]. For a POI to be attached to a protein-based scaffold, the Tag and the Catcher are fused to the N- or C-terminal of both proteins. Upon mixture, the proteins conjugate without the need for an intermediate, contrarily to CID and enzyme-mediated conjugation. The SpyTag/SpyCatcher reaction can proceed up to 90% efficacy on ferritin nanoparticles [59,60] and may occur in nearly any common conditions such as pH 5–10 and temperatures of 4–37 °C in the presence or absence of reducing agents [89].

**Table 3:** Surface functionalization of ferritin nanoparticles by the modular assembly for antigen-display applications.

Strategy	Expression System		Ferritin Source	Conjugate	No. Residues	Target	Architectonic Tags	Conjugation Mechanism	Attachment Region on Ferritin	Conjugation Efficiency	Application	Ref
	Ferritin	Conjugate										
Chemical crosslinking	Equine spleen (out-sourced)	(out-sourced)	Equine spleen	KGDS peptide	4	Human activated platelets	-	Activation of KGDS with EDC and NHS; conjugation of KGDS-NHS to NH_2_ moieties on ferritin	Solvent-accessible NH_2_ moieties	-	Cell targeting	[86]
Chemical crosslinking	Equine spleen (out-sourced)	HEK293 (out-sourced)	Equine spleen	HA	552	Influenza virus	-	NHS-PEG-Mal crosslinker (NHS arm reacts with NH_2_ moieties on ferritin; Mal arm reacts with C residues on HA)	Solvent-accessible NH_2_ moieties	-	Viral vaccination	[90]
Chemical crosslinking	*E. coli*	Mice	Human	IgG	(~150 kDa)	Melanoma	-	NHS-PEG-Mal crosslinker (NHS arm reacts with NH_2_ moieties on IgG; Mal arm reacts with C residues on ferritin)	Solvent-accessible C residues	-	Tumor targeting	[85]
CID	*E. coli*	*E. coli*	*P. furiosus*	eGFP	223	-	FKBP fused to eGFP; FRB fused to ferritin	Heterodimerization of FKBP and FRB by addition of rapamycin	N-terminal	-	Multivalent protein-protein interaction	[58]
Click-chemistry	*E. coli*	Mice	Human L-chain	IgG	(~150 kDa)	Cell adhesion molecule ICAM-1	4-AzF incorporated in res 5 of ferritin; IgG functionalized with DBCO (via DBCO-NHS)	Conjugation of DBCO to 4-AzF by click chemistry	N-terminal	< 57%	Cell targeting	[65]
Enzyme-catalyzed	*E. coli*	*E. coli*	Human H-chain	7D12	(~15 kDa)	EGFR^+^ A431cancer cells	Q residue fused to C-terminal of 7D12; KK residues and ferritin spaced by a (GS)_3_ linker	Transglutaminase mediates stable isopeptide bond between Q and K residues	Fused KK residues atN-terminal	-	Tumor targeting	[73]
Tag/Catcher	*E. coli* BL21(DE3)	*E. coli* BL21(DE3)	*P. furiosus*	E7 Reps1 Adpgk Dpagt1	21 28 28 25	E7-related or MC38 tumors	SpyTag fused to C-terminal of peptides; SpyCatcher and ferritin spaced by a (G_4_S)_3_ linker	Spontaneous isopeptide bond formation between SpyTag and SpyCatcher under nearly any common conditions	N-terminal	90%	Tumor targeting	[59]
Tag/Catcher	*E. coli* BL21(DE3)	*E. coli* BL21(DE3)	*P. furiosus* or mouse H-chain	preS1 of HBV	108	HBV	SpyCatcher fused to C-terminal of preS1; SpyTag and ferritin spaced by a (G_4_S)_3_ linker	Spontaneous isopeptide bond formation between SpyTag and SpyCatcher under nearly any common conditions	N-terminal	90%	Viral vaccination	[60]
Tag/Catcher	*E. coli* BL21(DE3)	HEK293F	*P. furiosus*	RBD of S protein	~220	SARS-CoV-2	SpyTag fused to C-terminal of RBD; SpyCatcher and ferritin spaced by a (G_4_S)_3_ linker	Spontaneous isopeptide bond formation between SpyTag and SpyCatcher under nearly any common conditions	N-terminal	-	Viral vaccination	[55]
Tag/Catcher	*E. coli* BL21(DE3)	CHO	*H. pylori*	RBD and HR of S protein	222 (RBD) 303 (HR)	SARS-CoV-2	SpyTag fused to N-terminal of RBD or HR; SpyCatcher fused to ferritin	Spontaneous isopeptide bond formation between SpyTag and SpyCatcher under nearly any common conditions	N-terminal	-	Viral vaccination	[40]
Tag/Catcher	CHO	CHO	-	RBD of S protein	202	SARS-CoV-2	SpyTag fused to C-terminal of RBD; SpyCatcher and ferritin; both spaced by a G_4_S linker	Spontaneous isopeptide bond formation between SpyTag and SpyCatcher under nearly any common conditions	N-terminal	-	Viral vaccination	[82]

4-AzF: unnatural amino acid 4-azidophenylalanine, allows site-specific conjugation of alkyne-containing small molecules (e.g., DBCO) or affinity ligands to the exterior surface of the nanocage; 7D12: anti-EGFR nanobody; E7 peptide: residues 43–62 of human papillomavirus 16 oncogene E7; eGFP: enhanced green fluorescent protein; FKBP: FK506 binding protein; FRB: rapamycin-binding domain of mTOR; HA: haemagglutinin of influenza virus; preS1: domain on hepatitis B virus (HBV) surface protein; IgG: immunoglobulin G; KGDS peptide—targets integrin GPIIb-IIIa located on the membrane of human activated platelets, associated with thrombosis; MC38: murine colon adenocarcinoma cell line; RBD and HR: receptor binding domain and heptad repeat, respectively, of spike (S) protein from SARS-CoV-2; Reps1, Adpgk, and Dpagt1—MC38 tumor-derived mutant neoantigens.

### 3.2. Attachment Site

As discussed above, some modular assembly strategies allow for a flexible selection of the position where the POI is attached to ferritin, while others and genetic fusion are restricted to the C- or N-terminals of ferritin. When this is the case, most studies opt to fuse the POI to the N-terminal of ferritin to prevent or at least reduce its impact on proper folding of the ferritin subunit, and also because the N-terminal faces the exterior of the nanoparticle. Yet, some antigens have been attached to loop regions spacing α-helices on the ferritin subunit. This display is ideally suited for small-sized antigens characterized by a hairpin conformation, such as the Fc binding peptide [56] and MtrE peptide loops from *N. gonorrhoeae* [38], because the antigens can keep their native conformation while their small size and location is unlikely to interfere with ferritin intersubunit interactions.

The functionalization of the C-terminal of ferritin has also been reported. Since the C-terminal faces the inner side of the nanoparticle, it is not surprising that it induces a weaker immune response compared to peptides displayed on the exterior surface [38]. Interestingly, Lee et al. [71] reported a recombinant turn-inside-out conformation after genetic fusion of a tumor antigen model at the C-terminal of ferritin, probably induced by the conformational flexibility of C-terminal E-helices on the ferritin subunits.

### 3.3. Linkers

Linkers may contribute to the expression efficiency, correct folding, and biological activity of the different proteins within the chimera. Determinant linker characteristics include size, amino acid composition, glycosylation status, and flexibility [91].

With respect to the peptide length, longer linkers better preserve the corresponding folding and biological activity of both the scaffold and the displayed proteins. In return, they may contribute to an increased likelihood of undesirable interactions between the displayed protein, and are more prone to cleavage by the host cell proteases [92], a matter worsened in eukaryotic cells containing complex proteases.

Typical linkers are repeats of glycine (Gly, G) and serine (Ser, S), and less frequently threonine (Thr, T) residues. These are uncharged, flexible, and hydrophilic small volume amino acids that contribute to reducing the likelihood of interference with the folding and biological activity of the proteins. In addition, the higher the content of Gly the more flexible the linker becomes [93]. Flexible linkers are associated with increased accessibility of the epitope, improved refolding [94], and higher *O*-glycosylation efficiency due to higher accessibility for glycosyltransferases to insert glycans.

*O*-glycosylation may occur in Ser- and Thr-containing linkers. It was found that xylose glycans were attached to Ser residues when GS linkers were used to express Fc-fusion proteins in CHO cells, and also on fibronectin-based fusion proteins produced in HEK cells [95]. Johnson et al. [96] reported that glycosylation in Ser residues may augment the fusion protein construct volume due to peptide elongation and less compaction of its backbone, and affect protein local conformation. Glycosylation also enhanced the rigidity of a proline-rich peptide linker [96].

Based on the discussion above, the optimization of linkers is thus important when designing fusion proteins in terms of both biological activity and conformation stability of the addressed proteins. Concerning ferritin-based antigen delivery systems, it is relevant to use a linker that enables the correct folding of the ferritin subunit and AOI so that proper assembly of the nanoparticle is achieved, and efficient immune response is triggered. Indeed, most of the ferritin fused to peptides/proteins has been expressed with GS-rich linkers up to fifteen residues in length, independently on the expression system, size/complexity of the antigen, and final application of the protein construct (in Architectonic tags, Table 2 and Table 3).

## 4. Production of Ferritin-Based Vaccine Candidates Using Recombinant Expression Systems

### 4.1. Expression Systems

The production of ferritin with genetically fused antigens/epitopes for vaccine or targeting purposes has been performed mainly in *E. coli* and HEK293 cells, while ferritin functionalized through modular assembly has been mostly expressed in *E. coli* (Table 2 and Table 3). Bacterial cells are unable to express glycosylated proteins and/or complex antigens with proper folding, forcing ferritin fusion constructs to be produced in animal cells. Table 2 summarizes highly glycosylated antigens (e.g., HA, gp140, S protein) fused to ferritin produced in mammalian cells. The know-how on the optimal cell and culture conditions for producing chimeric ferritin may as well affect the host organism of choice; most of the work employing CHO cells to produce ferritin-based vaccines has been developed by the same research group [79,80,82,84].

Although examples are scarce, ferritin nanoparticles can also be produced with a high degree of purity using insect cells and the baculovirus expression vector system (IC-BEVS) [42] thus offering an alternative platform for the production of ferritin-based vaccines. Chen et al. [43] produced a ferritin-based vaccine candidate against foot-and-mouth disease virus using *Sf*-9 cells; however, the vaccine-elicited partial protection in mice compared to the commercial inactivated vaccine, and it was reported that the expression system must be improved to increase the solubility of the final nanoparticles. More recently, the same research group added the signal peptide of the human CD5 leader sequence to efficiently guide the secretion of an E2 glycoprotein-ferritin nanoparticle targeting the classical swine fever virus (CSFV). Again, *Sf*-9 cells were selected to express this vaccine candidate, which was shown to induce an earlier production of neutralizing antibodies in immunized rabbits compared with the subunit counterpart [44].

### 4.2. Production of Ferritin Nanoparticles

For chimeric ferritin nanoparticles that result from genetic fusion, the production yield is influenced by the size of the fused epitope/antigen (i.e., number of residues). When small peptides of around ten amino acid residues in length are fused to the ferritin, the production yield is about 10-fold higher compared to when complex antigens are fused to the nanoparticle (Table 2). A considerable amount of cell energy resources is spent in assembling the complex antigens rather than in expressing ferritin nanoparticles, which translates into a decreased production yield. Together with the higher chance of improper folding, the antigen size-dependent yield is yet another drawback associated with the production of ferritin-based delivery systems when genetic fusion is used as a strategy for antigen attachment.

Some authors have reported increased production yield of complex ferritin-antigen nanoparticles by 20 to 40-fold when the chimeras were produced in CHO compared to HEK cells [53,80]. In addition, CHO-derived products presented higher homogeneity and better assembly of the nanoparticles [79], making this expression system an attractive platform to produce ferritin-based nanoparticles with fused complex antigens.

As aforementioned, one of the advantages of modular assembly is the possibility of producing ferritin and antigen separately in the respective optimal host cell, followed by conjugation of the protein parts. Ma et al. [40] reported a higher expression yield when ferritin was expressed in *E. coli* and the AOI—the receptor-binding domain (RBD) and heptad repeat (HR) of the S protein from SARS-CoV-2—were expressed in CHO cells, compared to when antigens were fused to ferritin nanoparticles and produced together in the same host.

Although modular assembly tends to result in higher production yields of each protein, the final yield will depend on the conjugation strategy. While methodologies like Tag/Catcher and enzyme-catalyzed conjugation allow for conjugation efficiencies of 90% [59,60] and 80% [88], respectively, chemical crosslinking and click-chemistry are not as appealing, with yields close or even lower than those obtained by genetic fusion. The most suitable upstream platform to produce a given ferritin-based vaccine candidate, including expression host, culture conditions, and ferritin-AOI attachment strategy, must thus be selected as a compromise that results in AOI and ferritin nanoparticles of good quality while retaining acceptable final production yields.

### 4.3. Purification of Ferritin Nanoparticles

**Figure 4:**
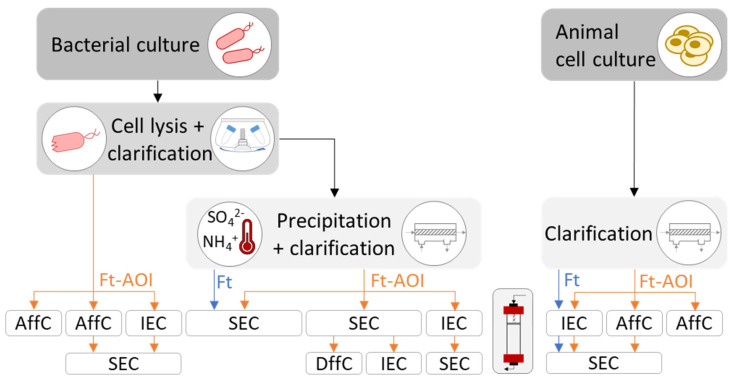
Strategies to produce and purify recombinant ferritin. AffC: affinity chromatography; DffC: differential centrifugation; Ft: ferritin; Ft-AOI: ferritin-antigen of interest; IEC: ion-exchange chromatography; SEC: size-exclusion chromatography.

Strategies employed to purify ferritin, native or outer surface-functionalized, are summarized in Figure 4. The decision depends mainly on the expression system (bacteria or animal cells) and the bioengineering strategies for ferritin functionalization (genetic fusion or modular assembly).

#### 4.3.1. Purification of Bacterial-Derived Ferritin Nanoparticles

The purification of recombinant ferritin nanoparticles produced in bacteria comprises, at an initial stage, cell disruption followed by clarification to discard debris. Taking advantage of ferritin’s thermostability, established protocols perform a heat treatment (around 70 °C for 10 min) of the clarified cell lysate to denature and precipitate up to 80% [97] of native *E. coli* proteins; this step may also be achieved via ammonium sulfate precipitation (65% saturation) [38,73]. Precipitated proteins are then separated by centrifugation, while soluble ferritin stays in the supernatant. Further purification steps comprising size-exclusion chromatography (SEC), and eventually differential centrifugation (DffC) or ion-exchange chromatography (IEC), yield high purity ferritin nanoparticles.

Some studies have bypassed the precipitation step of native *E. coli* proteins, which may be useful if the precipitation has deleterious effects on the antigen/epitope fused to the ferritin. Indeed, studies that have not performed this step were those involving a ferritin-AOI (Ft-AOI) chimera (Figure 4). In such cases, ferritin nanoparticles were separated either by affinity chromatography (AffC), using an inserted His-tag on the ferritin or its fused antigen [39,58,71], or by IEC followed by SEC up to 98% of purity [49,66].

For modular assembly, an extra purification stage is required after performing the conjugation step. Generally, SEC is employed to separate the Ft-AOI constructs of expected size from excess reagents and/or defective by-products resultant of ineffective conjugation such as aggregates and heterogenous oligomers. Ultracentrifugation can also be used, although with limited scalability.

#### 4.3.2. Purification of Animal-Derived Ferritin Nanoparticles

The purification of recombinant ferritin nanoparticles from animal cell culture is less complex compared to bacteria. The protein is first separated by clarification using either centrifugation or filtration and further purified by chromatographic approaches. In the presence of ferritin alone, IEC will suffice; if ferritin is genetically modified, then AffC can be used taking advantage of the binding capacity of the antigen attached to ferritin. Examples of AffC include agglutinin against influenza HA [45,47,50], lectin against HIV-1 gp140 [63,79] and SARS-CoV-2 S protein [83], a neutralizing antibody [78,79,80] against HIV-1 gp140, and a monoclonal antibody against hepatitis C virus (HCV) E2 glycoprotein [84]. Generally, SEC is performed as a final polishing step (Figure 4); nonetheless, purities above 95% were reported after performing a single affinity chromatographic step with a broadly neutralizing antibody or Ni-NTA to purify ferritin nanoparticles displaying HIV-1 gp140 trimers [78] or CSFV E2 glycoprotein [44], respectively.

Instead of using the binding capacity of the antigens, Jacob et al. [98] developed a novel method to purify ferritin-based vaccines by using the ferritin core as a target for affinity chromatography. Multicomponent Ugi ligands, with affinity to hydrophobicity sites on ferritin, were coupled to the resin; due to the presence of HA trimers on the surface of ferritin, a PEG-spacer arm was added to enable accessibility to the ferritin. This approach presents a potential universal method of purifying ferritin-based vaccines.

### 4.4. Nanoparticle Characterization

Ferritin nanoparticles can be quantified using standard methods of protein quantification. The colorimetric bicinchoninic acid (BCA) assay has been employed to quantify ferritin [40,72,86]. Another commonly used method is to measure the absorbance of the aromatic amino acids tyrosine and tryptophan that are present in the protein at 280 nm [68,85] based on the theoretical molar extinction coefficient of the ferritin nanoparticles, such as 4.56 × 10^5^ M^−1^cm^−1^ for human ferritin on a 24-mer basis [85].

Sodium dodecyl sulfate-polyacrylamide gel electrophoresis (SDS-PAGE), and less commonly native PAGE, can be used to separate proteins based on their molecular weight. Since SDS-PAGE operates under reducing conditions, ferritin is in monomeric form and weighs about 20 kDa, plus the weight of any POI fused or conjugated to it. In native PAGE, the ferritin nanoparticle is assembled and expected to weigh around 480 kDa, plus the weight of any POIs fused or conjugated to it.

For structural analysis, size-exclusion chromatography (SEC) is essential to confirm nanoparticle assembly and size. Dynamic light scattering (DLS) allows for measuring the size and dispersity of the nanoparticles, while transmission electron microscopy (TEM) confirms their shape. Circular dichroism (CD) is occasionally employed to assess the folding of both the ferritin and fused antigen. Less frequently used analytics include X-ray crystallography [38,52] and cryo-EM [37,50,52,81] to resolve the molecular structure of ferritin nanoparticles. Mass spectrometry (MS) has been applied to obtain the molecular weight of the Ft-AOI constructs [39,56] and to confirm the insertion of antigenic peptides [57]. Hydrophilic interaction chromatography (HILIC) was used to analyze the glycosylation profile of HIV trimers displayed on ferritin [9].

Biological activity, commonly assessed in terms of binding capacity and ability to induce an immune response, is critical for ferritin-based therapeutical applications. The binding capacity can be studied by means of quartz crystal microbalance (QCM) [56], surface plasmon resonance (SPR) [40,50,56], and biolayer interferometry [9,81]. To assess the immunogenicity of the ferritin constructs, antibodies induced upon administration of ferritin-based vaccines present on sera of immunized animals are commonly quantified with enzyme-linked immunosorbent assay (ELISA), virus neutralization assay, and flow cytometry.

## 5. Concluding Remarks

Ferritin nanoparticles are stable, biocompatible, and amenable to genetic fusion and chemical conjugation. In the last 15 years, ferritin has been engineered to serve as an antigen-display platform in the development of therapeutics, such as immunotherapy and vaccination.

Ferritin-based vaccines are safe and can be produced rapidly and at a low cost. Their potential to elicit immune responses has been demonstrated against a wide range of pathogens. Although recent, ferritin particles are already in Phase 1 clinical trials, three of which have started between the end of 2020 and the beginning of 2021, thus demonstrating the raising interest in this platform.

Novel vaccine formulations based on modular assembly would allow producing ferritin and antigens separately in their respective optimal host cell while maintaining native conformations. In addition, different antigens could be conjugated in the same nanoparticle, enabling the development of broad-spectrum vaccines. Finally, large stocks of ferritin could be easily stored and functionalized at a later stage in the event of an epidemic or pandemic, resulting in a versatile platform for producing safe vaccines in a rapid fashion.

A major challenge of ferritin-based vaccines will likely be related to antigen design, i.e., whether its structure can be maintained and/or if it is capable of inducing an immune response after conjugation to ferritin. The continuing evolution of computational tools will be essential for in silico prediction of acquired antigen conformations and thus aid the selection of preferred epitopes or the engineering of new epitopes. If conceiving modular assembly of different antigens to a single ferritin nanoparticle, the challenge will be to combine the required conjugation methods in such a way that there is no interference between them, and antigens can be attached in a controlled and standardized manner so that a homogenous final product is obtained. Further drawbacks related to ferritin-based products include the lack of control over subunit self-assembly and the difficulty in performing a precise and reliable analysis of the assembled products at the molecular level.

The implementation of a standardized method to produce and purify ferritin-based vaccine candidates would bypass the need to develop a novel protocol whenever ferritin is functionalized with a new antigen, facilitating the approval by the responsible regulatory agencies, and shortening the long process encompassed between the initial conceptualization of a vaccine and its licensing.

In conclusion, the development of ferritin-based vaccines holds great promise but will have to cope with associated challenges. Protein nanoparticle vaccines based on modular assembly are an exciting platform for flexible and secure vaccines.

## Figures and Tables

**Table 1:** Ferritin sources used for ferritin-based vaccine production, expression system, and putative *N*-glycosylation sites.

Ferritin Source	Uniprot	Expression System	Putative *N*-Glycosylation Site	Sequon ^1^	References
*E. coli* K-12	P0A998	*E. coli*	-	-	[37]
*H. pylori*	P52093	*E. coli*	Eukaryote and bacteria	E(17)-M(18)-N(19)-S(20)-S(21)	[38,39,40]
*H. pylori* (N19Q) ^2^	-	IC-BEVS, HEK293	-	-	[41,42,43,44,45,46,47]
*H. pylori*-bullfrog hybrid ^3^	-	*E. coli*, HEK293, CHO	-	-	[48,49,50,51,52,53]
*P. furiosus*	I6V0I9	*E. coli*	-	-	[54,55,56,57,58,59,60]
*P. furiosus* (R64K) ^4^	*-*	HEK293	-	-	[9]
*A. fulgidus*	O29424	*E. coli*	Eukaryote and bacteria	E(96)-V(97)-N(98)-V(99)-T(100)	[54,61,62]
*T. ni*	L-chain: Q52SA8 H-chain: Q52SA9	HEK293	Eukaryote (L-chain only)	L-chain: R(101)-K(102)-N(103)-Y(104)-T(105)	[63]
Rat	L-chain: P02793 H-chain: P19132	HEK293	Eukaryote (both chains) ^5^	L-chain: R(6)-Q(7)-N(8)-Y(9)-S(10) H-chain: S(110)-V(111)-N(112)-Q(113)-S(114)	[20,60]
Human	L-chain: P02792 H-chain: P02794	*E. coli*	Eukaryote (both chains) ^5^	L-chain: R(6)-Q(7)-N(8)-Y(9)-S(10) H-chain: N(110)-V(111)-N(112)-Q(113)-S(114) and S(179)-D(180)-N(181)-E(182)-S(183)	[16,64,65,66,67,68,69,70,71,72,73]

^1^ The eukaryotic *N*-glycosylation consensus sequon is N-*X*-S/T; the bacterial sequon requires a D/E at the -2 position, i.e., D/E-*Y*-N-*X*-S/T (where *Y* and *X* can be any amino acid except proline) [74]. Aminoacids colored in grey indicate the absence of the required D/E at position -2 characteristic of the bacterial sequon. ^2^ *H. pylori* nonheme ferritin (residues 5–167) with a point mutation at residue 19 (N19Q) to abolish a potential *N*-glycosylation site. ^3^ Fusion of residues 2–9 of the bullfrog (*R. catesbeiana*) L-chain ferritin (N8Q) to residues 3–167 of *H. pylori*. ^4^ *P. furiosus* ferritin with a point mutation at residue 64 (R64K) to eliminate a potential cleavage site. ^5^ Ferritin helices A and C are solvent-exposed, thus if a sequon is present in any of these then an *N*-linked glycosylation may occur. While consensus sequence N(8)-Y(9)-S(10) of the L-chain may lie on the outside of the assembled molecule [75], the consensus sequence on the H-chains may occur at an intersubunit interface [75], thus prohibiting *N*-glycosylation bond.

## Data Availability

Not applicable.
